# Polysplenia Syndrome and the Development of Heart Failure due to Associated Congenital Heart Defect

**DOI:** 10.5334/jbsr.3770

**Published:** 2024-12-13

**Authors:** Valerie Van Ballaer, Pieter Meersman, Nico Hustings

**Affiliations:** 1 Department of Radiology, UZ Leuven, Leuven, Belgium; 2 Department of Radiology, Sint-Franciscusziekenhuis, Heusden-Zolder, Belgium

**Keywords:** Polysplenia syndrome, Heterotaxy syndrome, Congenital heart defects, Congenital anomalies, Pulmonary hypertension, Right heart failure, Computed tomography (CT), Echocardiography, Magnetic resonance imaging (MRI)

## Abstract

*Key message:* Patients with polysplenia syndrome can develop pulmonary hypertension and heart failure due to underlying congenital heart disease, underscoring the need for early recognition and intervention to prevent further progression of the condition.

## Case Presentation

**Figure 1:**
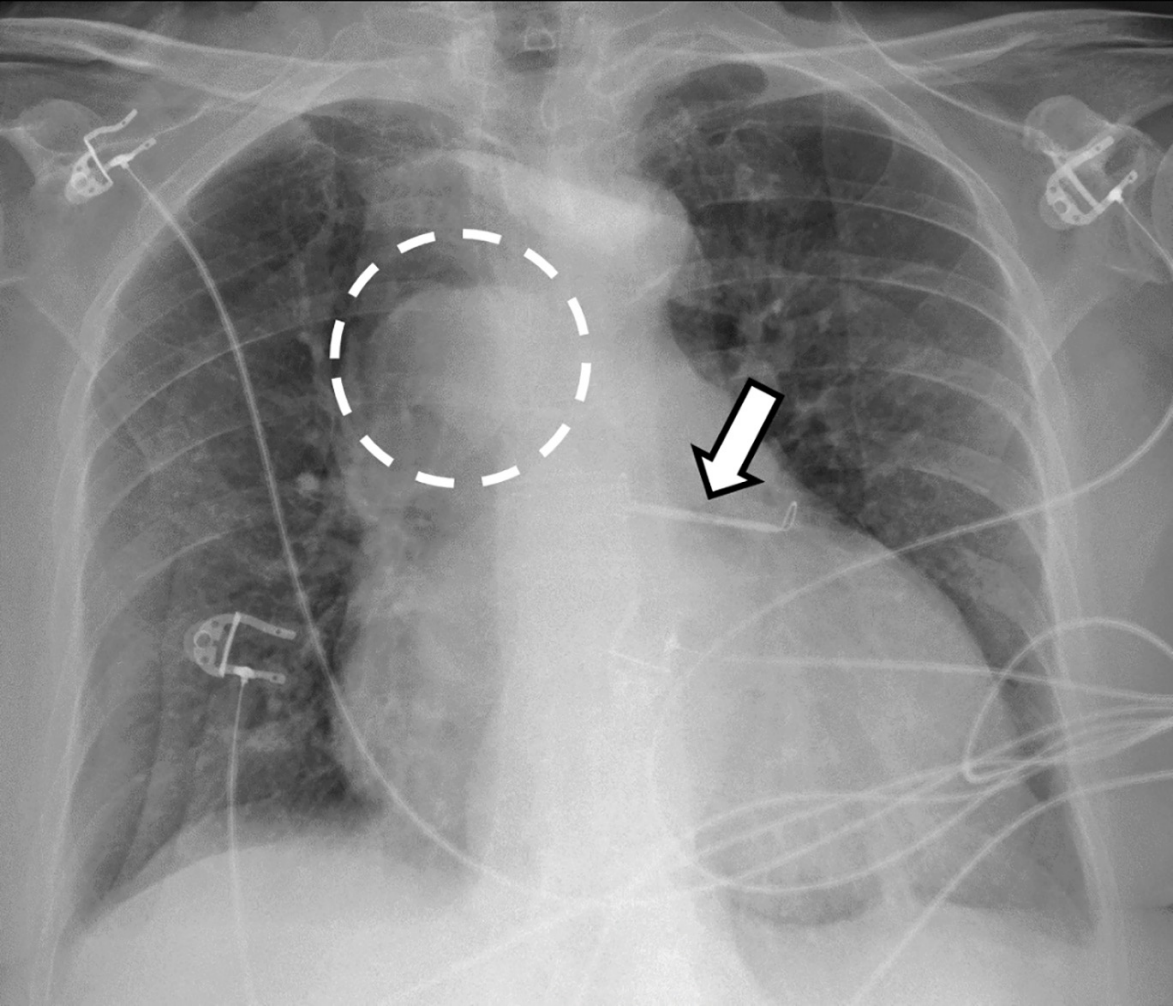
Chest radiograph showing cardiomegaly and a nodular right‑paratracheal opacity (dashed circle), suggestive of an enlarged azygos vein. A metallic opacity (arrow) from a previous clipping of the left atrial appendage is also visible.

A 65‑year‑old woman presented to the emergency department with complaints of shortness of breath and peripheral edema. A conventional chest radiograph revealed cardiomegaly and a nodular right‑paratracheal opacity (Figure 1, dashed circle), indicative of an enlarged azygos vein. In addition to electrocardiogram (ECG) monitoring, a metallic opacity (Figure 1, arrow), from a previous clipping of the left atrial appendage, is noted.

**Figure 2:**
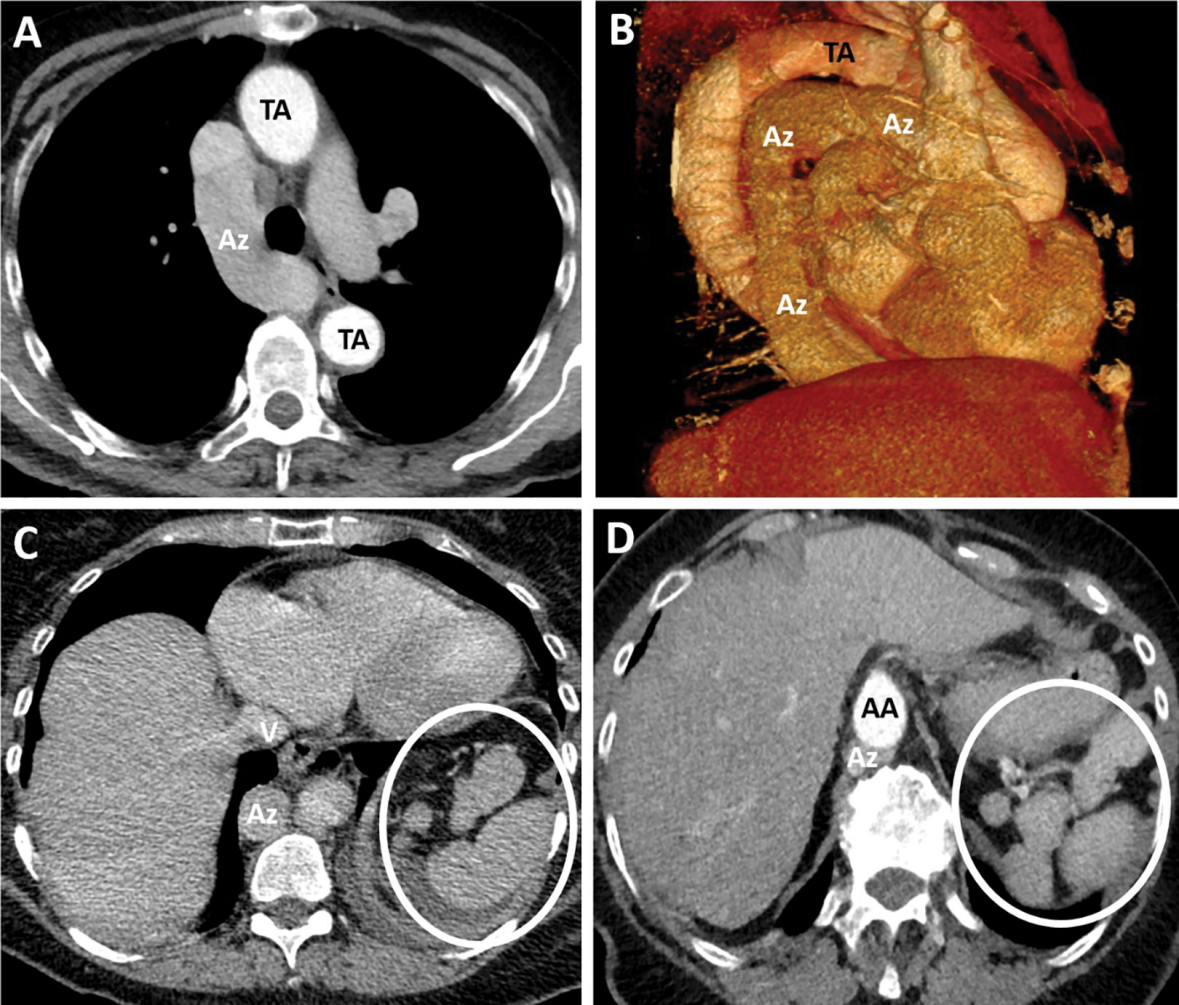
Thorax CT scan illustrating features of polysplenia syndrome. An azygos continuation of the inferior vena cava is noted (Az), with an enlarged thoracic azygos vein draining systemic veins. Polysplenia (white circle) is observed in the left subdiaphragmatic region alongside an aberrant midline abdominal aorta.

A subsequent thorax computed tomography (CT) showed features consistent with polysplenia syndrome, most notably, an azygos continuation of the inferior vena cava, in which an enlarged thoracic azygos vein (Az) continues infradiaphragmatically and drains all the systemic veins of the abdomen and lower extremities. The inferior vena cava (V) itself drains only the hepatic veins. Additionally, polysplenia was present in the left subdiaphragmatic region, along with bilateral bi‑lobar lungs and bilateral atrial appendages with left atrial morphology (not shown). While the thoracic aorta follows a normal course (TA), the abdominal aorta (AA) exhibits an aberrant midline course (Figure 2A–D), polysplenia represented by white circle.

**Figure 3:**
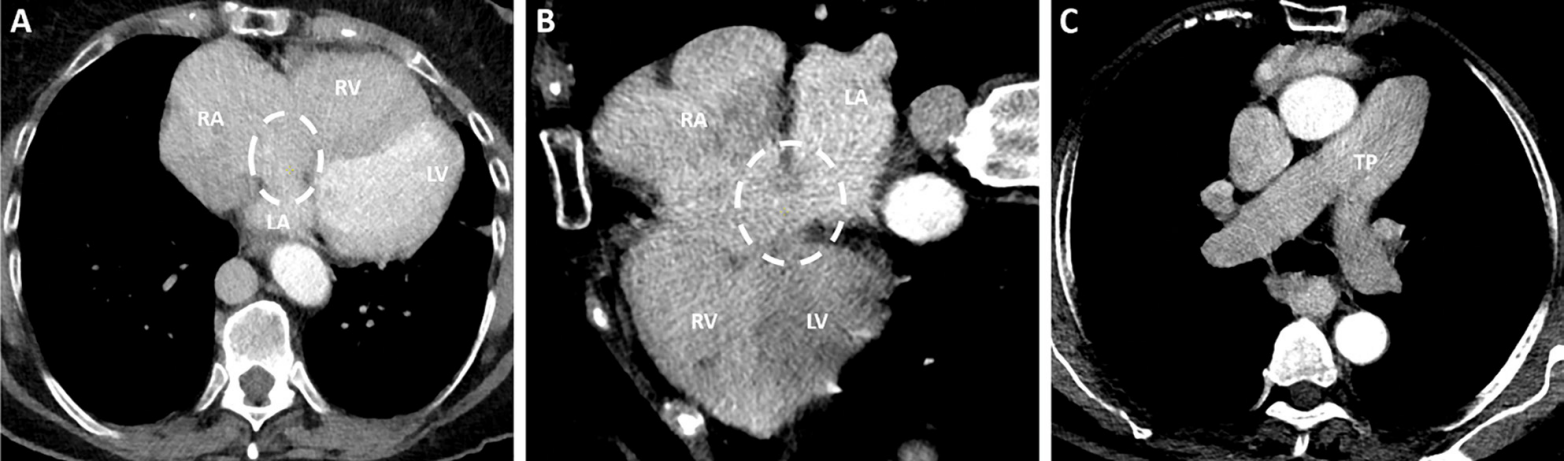
Imaging findings related to atrial septal defect (ASD) in polysplenia syndrome. A dilated pulmonary trunk (TP), right atrium (RA), and right ventricle (RV) are evident. The dashed circle indicates the congenital ASD, contributing to left‑to‑right shunting.

A significant component of the polysplenia syndrome in our patient, now causing her clinical symptoms, is a congenital atrial septal defect (ASD). This is associated with a dilated pulmonary trunk (TP), dilated right atrium (RA), and right ventricle (RV) (Figure 3A–C, ASD represented by dashed circle).

The constellation of cardiac and clinical findings suggests that ASD has led to chronic left‑to‑right shunting, increasing the preload of the right heart chambers, which in turn raises right‑sided pressures and, ultimately, causes pulmonary hypertension and right heart failure.

An additional echocardiogram (not shown) supports these findings, demonstrating right heart dilation, severe tricuspid valve regurgitation, and severe pulmonary hypertension, with only slight mitral valve regurgitation, normal left atrium (LA) and ventricle (LV) size with preserved left ventricular systolic function. Cardiac magnetic resonance imaging (MRI) (not shown) could be a noninvasive method for the assessment of the hemodynamic impact in this patient with ASD by quantifying the shunt fraction.

## Comment

Patients with polysplenia syndrome, also known as left isomerism, are characterized by symmetrical structures typically found on the left side of the body. [1] Common features include the absence or interruption of the inferior vena cava (IVC) with azygos continuation, multiple spleens (polysplenia), bilobed lungs, and bilateral left atrial morphology. Left isomerism is also associated with cardiac malformations, which tend to be milder compared with the more severe anomalies seen in right isomerism (asplenia). Specific congenital heart defects commonly observed in polysplenia syndrome include atrioventricular septal defects, ventriculoarterial disconnections, and abnormal venous return, such as anomalous pulmonary venous connections. Polysplenia syndrome is, thus, an independent risk factor for congenital heart disease‑associated heart failure. Early detection and timely intervention in patients with polysplenia syndrome and congenital heart disease are crucial to prevent heart failure and improve long‑term outcomes.
